# Visualizing the dynamic change of Ocular Response Analyzer waveform using Variational Autoencoder in association with the peripapillary retinal arteries angle

**DOI:** 10.1038/s41598-020-63601-8

**Published:** 2020-04-20

**Authors:** Shotaro Asano, Ryo Asaoka, Takehiro Yamashita, Shuichiro Aoki, Masato Matsuura, Yuri Fujino, Hiroshi Murata, Shunsuke Nakakura, Yoshitaka Nakao, Yoshiaki Kiuchi

**Affiliations:** 1 Department of Ophthalmology, Graduate School of Medicine and Faculty of Medicine, The University of Tokyo, Tokyo, 113-8655 Japan; 2 Seirei General Hospital, Shizuoka, 430-8558 Japan; 3 Seirei Christopher University, Shizuoka, 433-8558 Japan; 4 Kagoshima University Graduate School of Medical and Dental Sciences, Kagoshima, 890-0075 Japan; 5 Department of Ophthalmology, Graduate School of Medical Sciences, Kitasato University, Kanagawa, 252-0374 Japan; 6 Department of Ophthalmology, Saneikai Tsukazaki Hospital, Hyogo, 671-1227 Japan; 7 Department of Ophthalmology and Visual Science, Hiroshima University, Hiroshima, 739-8511 Japan

**Keywords:** Predictive markers, Refractive errors

## Abstract

The aim of the current study is to identify possible new Ocular Response Analyzer (ORA) waveform parameters related to changes of retinal structure/deformation, as measured by the peripapillary retinal arteries angle (PRAA), using a generative deep learning method of variational autoencoder (VAE). Fifty-four eyes of 52 subjects were enrolled. The PRAA was calculated from fundus photographs and was used to train a VAE model. By analyzing the ORA waveform reconstructed (noise filtered) using VAE, a novel ORA waveform parameter (Monot1-2), was introduced, representing the change in monotonicity between the first and second applanation peak of the waveform. The variables mostly related to the PRAA were identified from a set of 41 variables including age, axial length (AL), keratometry, ORA corneal hysteresis, ORA corneal resistant factor, 35 well established ORA waveform parameters, and Monot1-2, using a model selection method based on the second-order bias-corrected Akaike information criterion. The optimal model for PRAA was the AL and six ORA waveform parameters, including Monot1-2. This optimal model was significantly better than the model without Monot1-2 (p = 0.0031, ANOVA). The current study suggested the value of a generative deep learning approach in discovering new useful parameters that may have clinical relevance.

## Introduction

The prevalence of myopia is growing globally^1^. Myopia is an important risk factor for several ophthalmological disorders such as cataract^2^, glaucoma^3^, choroidal neovascularization^4^, and retinal detachment^5^, because of the associated structural changes. For example, the severity of myopic maculopathy increases with increased axial length (AL) and with decreased spherical equivalent refractive error (SERE)^6^.

In myopic eyes, the retina is mechanically stretched around the papillomacular bundle. This retinal deformation is demonstrated by the circumpapillary retinal nerve fiber layer (cpRNFL) peak angle (defined as the angle between the supratemporal and inferotemporal peak of the cpRNFL thickness profile) and by the peripapillary retinal arteries angle (PRAA)^7–9^. The range of individual variability in AL at birth is large^10^ and longer AL does not necessarily mean that the eye is elongated. In fact, even if two eyes have identical AL in adulthood, if they had different AL at birth the degree of elongation and the associated retinal stretch during the growth period are different between the two eyes^7^. Furthermore, we previously reported that AL may increase in adulthood^11^, in line with a previous paper^12^. In line with this, we previously reported that the correlation between AL and the cpRNFL peak angle (*r* = −0.49) or the PRAA (*r* = −0.38) was moderate^7^.

Ocular biomechanical properties can be measured by using the Ocular Response Analyzer (ORA; Reichert Inc., Depew, NY, USA) and Corvis Scheimpflug Technology (CST; Oculus, Wetzlar, Germany). By analyzing CST biomechanical parameters, we previously reported that the ability to absorb the applied external energy (hysteresis) was significantly associated with myopic retinal stretch as estimated by the cpRNFL peak angle^13^. Another study reported that the maximum deformation amplitude as measured by using CST was associated with the size of β-zone parapapillary atrophy^14^. With the ORA, it is possible to evaluate corneal biomechanical properties in detail by analyzing the recorded waveform rather than the single parameter of Corneal hysteresis (CH). For instance, we have reported that parameters extracted from the ORA waveform were more strongly correlated with glaucomatous visual field progression compared to CH^15^. By using this approach, we recently reported that the corneal biomechanical properties described by parameters extracted from the ORA waveform were significantly related to myopic retinal deformation^16^. Currently, in the ORA 37 parameters are shown, which are derived from the two ORA response wave peaks, as implemented by the manufacturer. However, in addition to these parameters, it is likely that analysis of the ORA waveform by means of advanced algorithms (e.g., machine learning) may be useful to capture further aspects of ocular biomechanics.

In machine learning, including deep learning, discriminative and generative models are used^17^. Several studies suggested the usefulness of a discriminative deep learning approach in Ophthalmology, for example for diagnosing glaucoma from a fundus photography^18–22^ and from optical coherence tomography (OCT)^23^. Variational Autoencoders (VAEs) are a type of deep learning approach that allows powerful generative models of data^24,25^. However, so far the usefulness of generative deep learning in Ophthalmology has not been assessed. VAEs consist of an encoder, a decoder, and a loss function. The input data is first processed using the encoder (a neural network), represented as a multidimensional probability density in a latent space, and then reconstructed by the decoder (a neural network). VAEs have demonstrated remarkable generative capacity and modeling flexibility, especially with imaging data^24^. Indeed VAEs have been used for various purposes, such as anomaly detection (for example, in Electrocardiograms^26^), clustering, and in particular, noise filtering^27^. Another feature of the VAEs approach is that it enables visualization of the dynamic change of input data by gradually shifting the latent variables, which may be helpful to understand its characteristics^24^.

The aim of this study was to assess whether possible changes in ORA waveform associated with changes in PRAA could be detected by using new ORA waveform parameters extracted from the ORA waveform by means of a VAE approach.

## Methods

### Study population

The study protocol was approved by the institutional review boards of the University of Tokyo Hospital, the University of Hiroshima Hospital, and the Tsukazaki Hospital and adhered to the tenets of the Declaration of Helsinki. Informed consent was obtained from each subject.

The sample of eyes included in the current study was the same as in our previous study^16^. ORA measurements were conducted in forty-nine normal eyes from 47 subjects. Inclusion criteria were: 1) no pathological findings by slit-lamp microscopy, ophthalmoscopy, and/or OCT; (2) best-corrected visual acuity ≤0.1 LogMAR (logarithm of the minimal angle of resolution); and (3) intraocular pressure ≤21 mmHg, as measured by using Goldmann applanation tonometry. Exclusion criteria were: (1) known ocular diseases such as glaucoma, staphyloma, and optic disc anomalies; (2) systemic diseases such as hypertension and diabetes; (3) the presence of visual field defects; and/or (4) a history of refractive or intraocular surgery, including cataract surgery.

### Measurements of AL and SERE

The AL was measured by using an optical biometer (OA-2000; Tomey, Nagoya, Japan). Three subsequent measurements were taken and the average value was used as AL measure. The SERE was measured by using the Topcon KR8800 autorefractometer/keratometer (Topcon, Tokyo, Japan).

### Peripapillary retinal arteries angle (PRAA)

**Figure 1:**
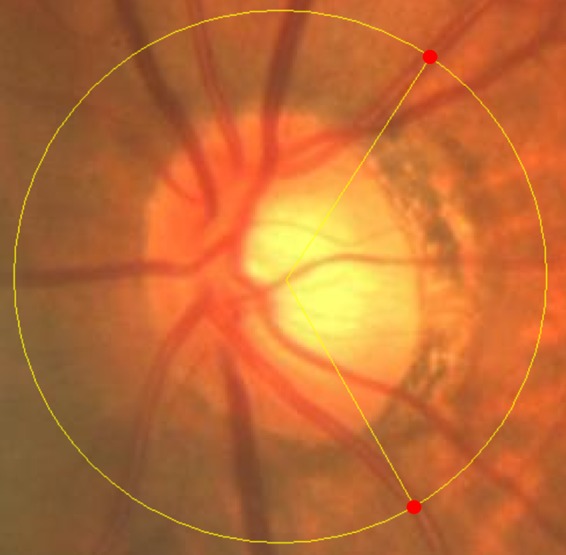
Measurement of peripapillary retinal arteries angle (PRAA) (left eye). The PRAA was calculated as the angle between the radia crossing the points (red dots) at the intersection between the 3.4 mm-diameter peripapillary scan circle (yellow) and the supratemporal/infratemporal major retinal arteries. The right eye was mirror-imaged.

The methodology to measure the PRAA has been reported in detail elsewhere^16^. Briefly, optic disc color fundus photographs were obtained by using either an OCT (OCT-2000®; Topcon, Tokyo, Japan) or a fundus camera (TRC-50DX®; Topcon). ImageJ software (https://imagej.nih.gov/ij/; provided by the National Institutes of Health, NIH, Bethesda, MD) was used to draw a 3.4-mm-diameter peripapillary scan circle on the obtained fundus photographs, and the PRAA was calculated as the angle between the radia crossing the points at the intersection between the 3.4 mm-diameter peripapillary scan circle and the supratemporal/infratemporal major retinal arteries (as shown in Fig. 1). Magnification effects of the camera were corrected by using the Littmann’s formula^28^.

### Analysis of the ORA waveform

The ORA waveform is recorded by using an electro optical system consisting of a collimated beam of infrared light that is reflected by the surface of the cornea onto a photodetector and represents the deformation of cornea (inward and outward movements) in response to a rapid air jet. The ORA waveform is composed of two peaks that correspond to the corneal inward and outward applanation events. The CH is defined as the difference in the two applanation pressure values and it represents the viscous damping of the corneal tissue^29^. The corneal resistant factor (CRF) is also calculated from the two applanation pressure values, but places greater emphasis on the first applanation pressure in order to give more information about the elastic properties of the cornea^29^. By using the ORA software (version 3.01), 37 waveform parameters can be obtained from the ORA waveform. The ORA waveform was recorded by using an ORA G3 model with the related PC software for waveform analysis. The ORA measurements were carried out three times with at least a 5-minute interval between each measurement, and the average of each obtained values were used in the analysis. All the measurement had a quality index of higher than 7.5 as recommended by the manufacturer.

### Variational autoencoder

**Figure 2:**
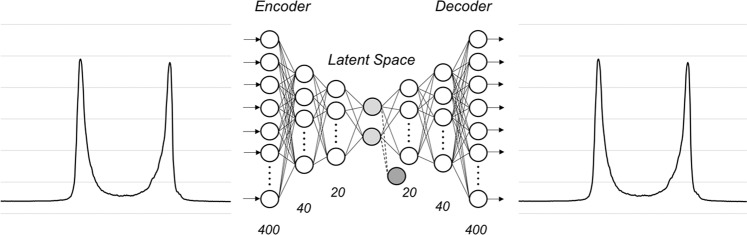
The VAE model implemented in this study. The input data has 400 dimensions, and this layer is connected to 2 hidden layers (light gray circles) with 40 and 20 dimensions, respectively (encoder). The encoder is connected to two dimensional gaussian distributions represented by the mean and logarithmic variance-covariance matrix in a latent space. The decoder reconstructs the ORA waveform data from the latent variables on 400 dimensions through 2 hidden layers with 20 and 40 dimensions respectively. VAE: variational autoencoder.

The structure of the VAE model used in this study is shown in Fig. 2. The encoder is a 1-layer neural network consisting of 400 units (one for each of the 400 ORA waveform observation points). This encoder is connected to 2 hidden layers consisting of 40 and 20 units, and is then represented by the mean and logarithmic variance-covariance matrix of a two-dimensional Gaussian probability density in the latent space. The decoder reconstructs the 400 units through a further 2 hidden layers and 1 output layer, which represents the reconstructed ORA waveform. This VAE model was optimized by maximizing the sum of the negative reconstruction loss obtained by using the current study’s dataset, which is defined as the Kullback–Leibler divergence between the distributions of the differences between the input ORA waveform and reconstructed ORA waveform. Then, the reconstructed ORA waveform was analyzed in conjunction with the PRAA.

### Identification of new ORA waveform parameter(s)

**Figure 3:**
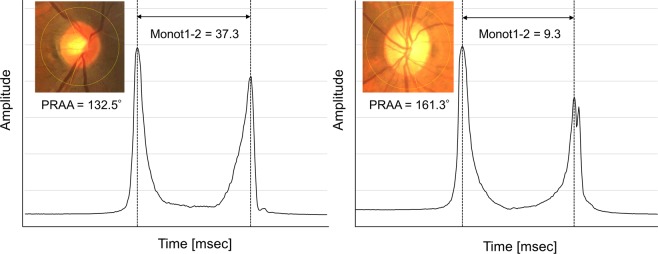
Measurement of Monot1-2 (left eye). Monot1-2 was estimated as the total length of retrogressive movement between applanation peak 1 and applanation peak. Left figure shows an example eye with long AL (51 years old, male, AL = 26.4 mm) with small PRAA (132.5°) and large Monot1-2 (37.3), whereas right figure is from a non-long eye (73 years old, female, AL = 23.44 mm) with large PRAA (161.3°) and small Monot1-2 (9.3). PRAA: peripapillary retinal arteries angle; AL: axial length.

By visual analysis of the changes in VAE-reconstructed ORA waveforms as a function of the changes in PRAA, we identified the change in monotonicity (the retrogressive movement) between the first and second applanation peaks (Monot1-2) as a possible new ORA waveform parameter potentially sensitive to changes in PRAA. More specifically, Monot1-2 was estimated as the total length of retrogressive movement between applanation peak 1 and applanation peak 2 (Fig. 3).

### Statistical analysis

The relationships between the PRAA and the 41 variables of age, AL, SERE, CH, CRF, 35 out of 37 ORA waveform parameters, and the newly proposed parameter Monot1-2 were evaluated by using a two-step feature selection approach in view of the high number of variables (41), following our previous study^16^. Two out of 37 ORA waveform parameters (i.e., h11 and h21) were not used as they are proportional to h1 and h2, respectively. First, 20 candidate variables were selected using the least absolute shrinkage and selection operator (Lasso) regression. Then, model selection was used to identify the optimal model for the PRAA by using the second-order bias-corrected Akaike information criterion (AICc) index from 2^20^ different patterns generated by using the 20 candidate variables. The Akaike information criterion (AIC) is a statistical measurement used in model selection^30^ and the AICc is a corrected version of the AIC, which is able to provide an accurate estimation even when the sample size is small^31,32^. A decrease in AICc indicates an improvement in the model^33^ and suggests that the variables selected through the model are significant^34^. It is worth noting that multivariate modeling such as the one here used can be useful for detecting patterns and characterizing data because it provides more control over potential confounders compared to univariate analysis^35^. The log-likelihood values of paired models (e.g., with vs without Monot1-2, multivariate vs univariate) were compared by using the analysis of variance (ANOVA) test.

All statistical analyses were performed by using R (version 3.4.3, http://www.R-project.org/).

## Results

The demographic characteristics of participants and of the ocular properties of the studied eyes are shown in Table 1.

**Table 1:** Characteristics.

Variables	Total	Hyperopia (SERE ≥ 0)	Mild myopia (−3 ≤ SERE < 0)	Moderate myopia (−6 ≤ SERE < 3)	High myopia (−6 ≥ SERE)
	Mean ± SD (Range)	Mean ± SD (Range)	Mean ± SD (Range)	Mean ± SD (Range)	Mean ± SD (Range)
Participants	52	17	17	12	6
Eyes	54	19	17	12	6
Age (y)	51.0 ± 21 (24–85)	71.0 ± 12 (29–85)	44.4 ± 18 (28–83)	39.4 ± 16 (26–71)	29.0 ± 5 (24–39)
Sex (male/female)	29/25	10/9	8/9	5/7	4/2
Axial length (mm)	24.6 ± 1.7 (21.5–28.1)	23.4 ± 1.0(21.5–25.7)	24.0 ± 0.9 (22.6–25.8)	25.9 ± 0.9 (24.8–27.1)	27.6 ± 0.4 (27.1–28.1)
Spherical equivalent (diopter)	−1.91 ± 4.1 (−12.6 to 4.1)	1.84 ± 1.2 (0 to 4.1)	−1.24 ± 0.8 (−2.9 to −0.1)	−4.41 ± 0.9 (−5.8 to −3.3)	−10.8 ± 1.5 (−12.6 to −9.0)
Keratometry (mm)	8.2 ± 0.5(7.4–9.2)	8.6 ± 0.3 (7.9–9.2)	7.9 ± 0.4 (7.4–8.6)	7.8 ± 0.3 (7.5–8.3)	7.7 ± 0.4 (7.4–8.3)
PRAA (degrees)	134.5 ± 14.8 (99.3–172.0)	140.4 ± 15.1 (112.8–172.0)	138.6 ± 14.3 (99.3–161.5)	123.8 ± 15.8 (105.6–156.0)	125.2 ± 17.3 (102.3–149.5)

SERE: spherical equivalent refractive error; SD: standard deviation; PRAA: peripapillary retinal arteries angle.

The change of the reconstructed ORA waveform using VAE associated with the decrease of PRAA is shown in Supplementary Video 1. Table 2 shows the summary descriptive statistics of the ORA parameters. Table 3 shows the results of univariate and multivariate linear regression between PRAA and the values of age, AL, CH, CRF, SERE, and ORA waveform parameters. AL and SERE were significantly correlated to the PRAA with univariate analysis (coefficient = −3.67, p = 0.0070, and coefficient = 1.70, p = 0.0022, respectively).

**Table 2:** Summary descriptive statistics of ORA parameters.

Variables	Mean ± Standard Deviation (Range)
CH (mmHg)	10.26 ± 1.0 (8.24–12.94)
CRF (mmHg)	9.85 ± 1.3 (7.20–12.33)
Aindex	9.96 ± 0.18 (8.78–10)
Bindex	9.70 ± 0.68 (5.69–10)
p1area	7441.74 ± 1693.32 (3409.38–12026.31)
p2area	5784.99 ± 1335.26 (3482.25–8540.52)
aspect1	26.10 ± 3.27 (17.21–31.98)
aspect2	22.56 ± 4.17 (13.78–30.78)
uslope1	82.54 ± 12.93 (52.51–112.77)
uslope2	81.18 ± 17.74 (42.61–132.66)
dslope1	39.17 ± 5.32 (26.44–51.25)
dslope2	32.69 ± 6.19 (18.98–43.02)
w1	23.73 ± 2.85 (18.67–32.67)
w2	24.14 ± 3.22 (18.67–32.00)
h1	611.54 ± 63.00 (371.00–673.38)
h2	526.29 ± 73.74 (359.63–652.75)
dive1	571.13 ± 61.44 (345.58–640.58)
dive2	478.27 ± 80.03 (278.25–631.67)
path1	16.43 ± 2.49 (10.29–21.26)
path2	18.32 ± 3.13 (13.39–32.33)
mslew1	156.73 ± 29.65 (76.83–213.42)
mslew2	135.12 ± 29.53 (84.00–207.17)
slew1	82.60 ± 12.85 (52.51–112.77)
slew2	81.99 ± 17.44 (47.29–132.66)
Aplhf	0.81 ± 0.23 (0.5–2.17)
p1area1	3697.37 ± 987.61 (1629.58–6567.63)
p2area1	2687.77 ± 702.61 (1457.50–4165.58)
aspect11	29.45 ± 4.42 (19.19–36.73)
aspect21	27.99 ± 5.79 (18.87–46.92)
uslope11	71.97 ± 13.36 (46.74–100.47)
uslope21	73.16 ± 15.18 (42.36–106.00)
dslope11	47.64 ± 7.20 (30.07–60.96)
dslope21	44.45 ± 9.29 (28.92–72.57)
w11	14.18 ± 2.30 (10.67–21.67)
w21	13.20 ± 2.18 (7.33–19.67)
h11	407.69 ± 42.00 (247.33–448.92)
h21	350.86 ± 49.16 (239.75–435.17)
path11	20.80 ± 3.85 (12.02–27.47)
path21	24.48 ± 3.94 (16.65–35.04)
Monot1–2	31.55 ± 25.97 (9.33–186.00)
Quality Index	8.81 ± 0.6 (7.55–9.70)

ORA: Ocular Response Analyzer; CH: corneal hysteresis; CRF: corneal resistance factor.

**Table 3:** Results of univariate and multivariate linear regression between PRAA and the values of age, AL, CH, CRF, SERE, and ORA waveform parameters.

Variables	Univariate analysis			Multivariate analysis			
	Coefficient	Standard Error	*P* Value	AICc	Coefficient	Standard Error	*P* Value
Age	0.16	0.11	0.16	459.1	−0.61	0.34	0.10
AL	−3.67	1.30	0.0070*	448.7	−0.32	5.35	0.95
CH	−3.33	2.24	0.15	453.4	3.19	5.63	0.58
CRF	−3.71	1.82	0.046*	451.5	−3.91	4.40	0.93
SERE	1.70	0.53	0.0022*	448.3	1.95	2.04	0.36
Aindex	4.95	6.44	0.56	452.4	17.16	24.11	0.49
Bindex	−5.34	3.25	0.11	452.0	−18.59	13.32	0.19
p1area	−0.0026	0.0013	0.06	466.4	0.056	0.056	0.34
p2area	−0.00099	0.0017	0.56	469.1	−0.018	0.038	0.64
aspect1	1.48	0.60	0.028*	451.9	−9.25	20.98	0.67
aspect2	−0.54	0.52	0.30	456.9	−5.25	8.68	0.56
uslope1	0.53	0.17	0.0025*	450.9	−9.15	12.86	0.49
uslope2	−0.16	0.13	0.20	459.3	−0.78	1.79	0.67
dslope1	0.57	0.34	0.14	456.1	5.64	6.37	0.39
dslope2	−0.17	0.34	0.62	458.6	2.21	3.92	0.58
w1	0.54	0.77	0.49	456.9	4.29	17.23	0.81
w2	0.79	0.66	0.25	456.1	−1.09	3.59	0.98
h1	0.13	0.031	0.00008*	447.8	0.48	0.68	0.49
h2	−0.027	0.031	0.38	463.0	0.25	0.41	0.56
dive1	0.13	0.033	0.0002*	449.3	−0.35	0.33	0.31
dive2	−0.026	0.029	0.37	463.1	−0.099	0.15	0.52
path1	−0.79	0.93	0.40	456.1	14.21	13.32	0.31
path2	0.054	0.71	0.94	457.4	3.03	8.17	0.72
mslew1	0.16	0.075	0.042*	457.7	−0.14	0.21	0.53
mslew2	−0.016	0.076	0.83	461.8	0.18	0.45	0.70
slew1	0.54	0.17	0.0024*	450.8	10.34	12.71	0.43
slew2	−0.11	0.13	0.41	460.1	0.31	2.10	0.89
Aplhf	−0.80	9.06	0.93	452.3	68.33	54.67	0.24
p1area1	0.0039	0.0023	0.097	466.0	−0.094	0.09	0.34
p2area1	−0.0029	0.0031	0.36	467.4	−0.007	0.07	0.92
aspect11	1.17	0.46	0.018*	452.2	−3.66	12.02	0.77
aspect21	−0.10	0.40	0.80	458.5	−4.56	7.33	0.55
uslope11	0.51	0.16	0.0025*	450.9	4.25	1.30	0.75
uslope21	−0.0034	0.14	0.98	460.5	1.91	1.08	0.10
dslope11	0.46	0.28	0.12	456.6	−1.28	3.24	0.70
dslope21	0.046	0.25	0.85	456.5	2.55	2.32	0.29
w11	0.11	0.93	0.91	456.9	−9.31	30.57	0.77
w21	0.36	1.04	0.73	456.5	13.94	10.60	0.21
path11	−0.69	0.61	0.27	456.5	−7.46	10.07	0.47
path21	0.48	0.54	0.38	457.1	−4.25	4.78	0.39
Monot1–2	−0.013	0.089	0.88	461.5	−0.78	0.42	0.09

*P value <0.05, AL: Axial length; CH: corneal hysteresis; CRF: corneal resistance factor; SERE: spherical equivalent refractive error.

The optimal linear model for the PRAA was identified as follows; PRAA = 238.9 – 2.37 (Standard Error: SE = 0.99, *p* = 0.021) × AL – 12.8 (SE = 4.00, p = 0.0069) × Bindex – 0.91 (SE = 0.41, p = 0.33) × aspect2 - 0.16 (SE = 0.089, p = 0.080) × mslew1 + 0.17 (SE = 0.042, p = 0.0003) × h1 + 0.44 (SE = 0.15, p = 0.0053) × uslope11 - 0.31 (SE = 0.11, p = 0.0069) × Monot1-2 (AICc = 433.7). The log-likelihood of the optimal model was significantly higher than that of the model without Monot1-2 (AICc = 435.6, p = 0.0031, ANOVA), and that of the univariate AL-only model (AICc = 448.7, p < 0.0001, ANOVA).

## Discussion

In the current study, we assessed the dynamic change of the ORA waveform in relation to retinal deformation as estimated by PRAA in a sample of 49 eyes from 47 participants, using VAE approach, a deep learning generative model. This study highlighted that a novel parameter extracted from the ORA waveform (Monot1-2) may be used to generate multivariate models of the PRAA that are more accurate than models without Monot1-2.

The optimal model for the PRAA in the previous study was as follows: PRAA = 68.6 – 3.0 × AL - 3.1 × CRF + 9.5 × Aindex + 1.8 × w2 + 0.40 × slew1, when the new ORA waveform parameter of Monot1-2 was not included^16^. Such waveform parameters represent a quick response of the cornea to external forces and suggest a soft cornea^36,37^. In the current study, eyes with high myopia were added, and the optimal model for PRAA was PRAA = 238.9 – 2.37 (Standard Error: SE = 0.99, *p* = 0.021) × AL – 12.8 (SE = 4.00, p = 0.0069) × Bindex – 0.91 (SE = 0.41, p = 0.33) × aspect2–0.16 (SE = 0.089, p = 0.080) × mslew1 + 0.17 (SE = 0.042, p = 0.0003) × h1 + 0.44 (SE = 0.15, p = 0.0053) × uslope11–0.31 (SE = 0.11, p = 0.0069) × Monot1–2. In the current study, AL was negatively associated with PRAA, similarly as in our previous study^16^. Besides, the ORA waveform parameters selected in the current study indicated that decrease in the size of the area of applanation (peak1, aspect2, uslope11, and h1), increase in maximum single length of the outside line segments of peak1 (mslew1), and increased lability in restoration phase/peak2 (Bindex) were related with smaller PRAA^36^, which infer quick cornea response and soft cornea behavior in line with our previous study^16^. In addition to these parameters, the current study showed that larger Monot1-2 was significantly related to smaller PRAA. This might be because eyes with smaller energy dissipation might have a greater amount of stored elastic energy during applanation 1 and 2, causing an increase in Monot1-2. Thus, results of the current study also seem to suggest eyes with greater myopic retinal deformation may demonstrate decreased energy dissipation.

The previous reports show that small energy dissipation of an eye represented by CH is related to rapid progression of glaucoma^38–40^. This may be because of similar biomechanical properties of the cornea and sclera^41^, as they are made up of the same types of collagen^42^. In addition, eyes experience great changes in the intraocular pressure even in daily life events, such as postural change^43^, eye lid blinking^44^, ocular pulsatility due to ocular hemodynamics^45^, Valsalva maneuver^46^, and eye movements^47^. Reduced energy dissipation in the cornea may cause higher vulnerability to these stresses^41^. Furthermore, we recently reported that angioid streaks were significantly associated with the corneal biomechanical properties as measured by using CST^48^. Considering these facts, the newly proposed Monot1-2 may also be useful in these diseases, and this should be further investigated in a future study.

A limitation of this study is the cross-sectional design. Further validation would be needed by using a longitudinal research approach, in particular in young population. We investigated the relationship between ORA waveform parameters and PRAA; however, subanalyses in association with the severity of myopia were not carried out due to the sample size in the current study. Further investigation is preferable with the larger population.

In conclusion, in this study a new ORA waveform parameter was proposed by analyzing, by using VAE, the dynamic change of the ORA waveform in relation to retinal deformation as estimated by PRAA. This was the first study to demonstrate the value of generative deep learning models such as the one generated by VAE, in discovering new useful parameters that may be helpful in the clinical setting.

## Supplementary information

Supplementary Video 1.

.
