# Supplementary material for: Visualizing the dynamic change of Ocular Response Analyzer waveform using Variational Autoencoder in association with the peripapillary retinal arteries angle
Source: Sci Rep. 2020 Apr 20;10:6592. doi: 10.1038/s41598-020-63601-8 (PMC7170838; doi:10.1038/s41598-020-63601-8)
Supplement: Supplementary file 2 — . [file 41598_2020_63601_MOESM2_ESM.docx]

Visualizing the dynamic change of Ocular Response Analyzer waveform using Variational Autoencoder in association with the peripapillary retinal arteries angle

Shotaro Asano^1^, Ryo Asaoka^1,*^, Takehiro Yamashita^2^, Shuichiro Aoki^1^, Masato Matsuura^1,3^, Yuri Fujino^1,3^, Hiroshi Murata^1^, Shunsuke Nakakura^4^, Yoshitaka Nakao^5^, Yoshiaki Kiuchi^5^

1: Department of Ophthalmology, Graduate School of Medicine and Faculty of Medicine, The University of Tokyo, Tokyo, 113-8655, Japan

2: Kagoshima University Graduate School of Medical and Dental Sciences, Kagoshima, 890-0075, Japan

3: Department of Ophthalmology, Graduate School of Medical Sciences, Kitasato University, Kanagawa, 252-0374, Japan.

4: Department of Ophthalmology, Saneikai Tsukazaki Hospital, Hyogo, 671-1227, Japan,

5: Department of Ophthalmology and Visual Science, Hiroshima University, Hiroshima, 739-8511, Japan,

* Correspondence and reprint requests to:

Ryo Asaoka,

Email: [rasaoka-tky@umin.ac.jp](mailto:rasaoka-tky@umin.ac.jp)
